# Laparotomy for Peritonitis

**Published:** 1889-06

**Authors:** 


LAPAROTOMY EOR PERITONITIS.—Lawson Tait claims to have cured six out of eight cases of acute suppurative peritonitis by laparotomy and drainage.

